# Report of the Committee on Practical Medicine

**Published:** 1857-07

**Authors:** F. R. Payne

**Affiliations:** Marshall, Illinois


THE NORTH-WESTERN

MEDICAL AND SURGICAL JOURNAL.

(NEW SERIES.)

VOL. VI. JULY, 1857. NO. 7.

### ORIGINAL COMMUNICATIONS.

ARTICLE I.—*Report of the Committee on Practical Medicine.* By F. R. PAYNE, Marshall, Illinois. Presented to the Æsculapian Society, at the meeting in Charleston, Ill., 27th and 28th of May, 1857.

Your reporters respectfully submit an imperfect sketch of some of the prevailing diseases of this district during the past year.

The duties of your committee are not only irksome, but difficult. About one hundred physicians have been addressed by circular, and six of that number have deigned to reply, and some of those in a very imperfect manner. It will be the object of your committee to present practical facts, based exclusively upon the experience and observation of practising physicians.

However pleasing to the mind it may be to theorize and speculate upon the etiology of disease, it is very evident by so doing our stock of medical knowledge would not be increased.

In order to build up and make still more respectable the profession of our choice, we must begin at the foundation, and sustain that department in which alone a suffering world is interested.

To the hospitals and the bedside of the sick we must go, in order to get a truthful history of disease, as we cannot by induction explain a symptom or point to a remedy.

During the last year, malarious diseases, the most common in this country, did not prevail to any considerable extent, the unmistakable cause of which all observing physicians fully understand. The season was very dry. The year preceding was extremely wet, and malarious diseases prevailed extensively among all classes and in all localities. During the winter months of 1855 and 1856, we had but little sickness; the weather was exceedingly cold, the thermometer varying from zero to 22° below. In the spring of that year "PNEUMONIA" prevailed, and was unusually severe and fatal. Dr. York, of Paris, says, "It has been much more fatal than at any former period in this part of Illinois." Dr. S. W. Thompson says, "The attacks were mostly complicated with gastro-enteritis. This was more specially the case in *Typhoid pneumonia*, which prevailed to a considerable extent last winter and spring."

Dr. T. D. Washburn says, "During eight years' residence in the Wabash Valley, I have never known sickness so general as in 1855. No locality seemed exempt. Many who were born and raised in Illinois, and had reached middle life without ever having had the ague, were this season made to quake like an aspen leaf."

Dr. Washburn states that "Pneumonia has been more fatal than usual the last year, 1856." He and Dr. York both concluded that the cause of this greater fatality was the depressing effects of malaria the previous fall, and excessive cold during the winter.

Dr. O. J. Herrick, of Midway, Ill., says that pneumonia has been much more fatal than usual the last year, and that a large proportion of his cases were complicated with bronchitis.

Dr. N. S. Holmes informs me that the cases in his locality were much more fatal than usual. He practises in the east part of this county.

From all the evidence your reporters have been able to obtain, they feel confident that pneumonia, in the spring of 1856, assumed a much more malignant form than in former years. Many of the symptoms of the disease, as presented in ordinary seasons, were greatly aggravated. In several fatal cases that came under our own observation, the expectoration of blood amounted almost to a hemorrhage; and we had, at the same time, great prostration and loss of vital energy, showing typhoid complication. Some died in the first or congested stage, but generally death occurred on the seventh or eighth day. If there was no amendment on the seventh day, the cases usually terminated fatally.

The symptoms did not vary from those laid down by our standard works, except in *degree.* The pulse ranged from 120 to 160 per minute, and at an early day, after the disease was fully developed, we had *subsultus tendinum*, delirium and partial cutaneous insensibility, indicative of much nervous derangement.

Last winter was very cold, but in the spring our cases of pneumonia were not as malignant as in the spring of 1856; but we have had more cases of phthisis among native citizens than ever before known in this latitude. During the last six months I have treated five well marked cases of tuberculoses, some of which are dead, and the others cannot recover.

It is the opinion of your reporters, that if our winters continue as cold as the two last, phthisis will become a common disease in this latitude.

TREATMENT.

Dr. York says, "The most successful treatment with which I am acquainted consists in the early and prompt administration of quinine, camphor and opium, in full doses, with one or two full doses of mercurials at the commencement, using, at the same time, mustard plasters freely to the side, spine and extremities. This course I continue until the systemic congestion is broken up, and the paroxysmal character of the disease is arrested; after this, I give *Pul. Doveri* every two or three hours, to allay pain and promote diaphoresis, and equal parts of tinct. sanguinaria, comp. syrup squills, and camph. tinct. opii, given every three or four hours in drachm doses. Blisters were applied to the seat of pain, generally within the first three or four days. In some cases it became necessary to use alterative doses of mercury during the whole course of the disease, and to resort to the early and prompt use of brandy and decoction rad.

senega. Great dyspnœa frequently occurred about the fifth or seventh day, for the relief of which I know of no means so successful as the application of large and strong mustard plasters along the spine and the insides of the thighs. This constituted the principal treatment in my practice."

Dr. S. W. Thompson says, "The enteric complication in a great measure precluded the use of tart. antimony. The treatment upon which I placed the most reliance consisted of small doses of calomel and morphine, with full doses of nitrate potas. and as much *Ipecac.* as the stomach would tolerate, given every two or three hours. Stimulating expectorants, as squill and senega, with early counter-irritation by means of blisters, and during the latter stages, quinine and *pul. dov.* with turpentine, in small doses, drops 10 and 15, every four hours. In some cases occurring amongst the old and feeble, and which had progressed to the stage of purulent infiltration, I found the cod liver oil, along with quinia and wine, to act most admirably."

Dr. T. D. Washburn makes the following statements: "The treatment in this locality varies somewhat. A few use the lancet, but *more* I think do not; some use quinine largely, and they affirm with much success; while others seldom use it in any stage of the disease. An active, mercurial cathartic is generally indicated at the outset, succeeded by *nauseating* doses of *tart.* antimony, or antimony and *Ipecac.* combined. If this is not well borne, I combine it with laudanum. Oftentimes I find the disease under this treatment arrested, or so much modified by the third or fourth day, that gentle diaphoretics and careful dietetic regulations for a few days will complete the cure.

"Venesection is frequently demanded; in the young and robust, who have a bounding pulse and tumultuous action of the heart, it should be pushed to syncope in the first stage of the disease. The cases that do not give way by the fourth day, I find, receive much benefit from a large blister over the seat of the disease. When expectoration is too profuse, and I have a constantly moist surface to contend with, I use the turpentine emulsion, combined with cinnamon, camphor and laudanum, and occasionally quinine. I never use quinine in this disease, except in the latter stage, unless there is a distinct intermission in the fever."

Dr. O. J. Herrick says that he is fully satisfied that the antiphlogistic treatment of pneumonia is wrong in this country. He resorts to the use of quinine early in the disease with satisfactory results.

This is a disease that is usually positively diagnosed, and our treatment ought to be equally positive and satisfactory. Experience and careful observation will make it so, if we seek for truth, and nothing but truth. The pathology being fully understood, and the diagnosis positive, careful observation and experience will wipe out all discrepancies in treatment, and make its therapeutics equally satisfactory. Even now, we find the variations in treatment are generally referred to the complication, and not to an imperfect knowledge of the disease itself.

In the Wabash Valley we frequently find pneumonia existing in a patient who is laboring under the influence of miasmatic poison, thus giving us a satisfactory explanation for the good effects of quinine; but we do not presume that those who understand the therapeutical properties of quinine will contend that it will control an inflammation. For what purpose, then, is it given in the first and second stages of this disease? not to cure the lungs, but to neutralize the poison that may be in the system, and thus remove a complication. This is a grave malady, and frequently destroys life in spite of medical skill. From thirty years of age, onwards, the mortality increases, and the previous occurrence of the disease greatly increases the danger. We have never known a case recover from the fifth attack, and but few survive the fourth. The treatment must be accommodated to the different stages and the disorders with which it may be complicated. In the congestive stage we apply sinapisms to the epigastrium, dorsal spine and extremities, and give internally sub. muriate 8 grs., ipecac. 3 to 6 grs., one powder every hour until two or three doses are taken. In six or eight hours after last powder, we give oil or senna and salts. After this operates, if there is remission in the fever, we give quinine and opium. We use as an expectorant *tinct.* sanguinaria and liquorice. In the stage of inflammation we use a solution of *tart. emetic* in *spts.* nitre, and give from two to four powders of calomel, *ipecac.* and opium, every twenty-four hours, and apply a large blister to the seat of pain. If we are satisfied that there is a complication, with remittent fever, which is evinced by a regular, well marked remission in the fever, we use quinine in each remission. In the stage of purulent infiltration, we give a stimulating expectorant, composed of senega squill and carb. ammonia, also quinine and wine.

Our limited experience in the use of veratrum viride, induces us to believe that it will prove an invaluable remedy in the treatment of this disease.

### TYPHOID FEVER.

From all the evidence your committee have been able to obtain, it seems to be rendered certain that typhoid fever, or a disease closely allied thereto, prevails to a considerable extent in the Wabash Valley. During a practice of sixteen years in this county, your reporter, until within the last five or six years, did not believe that this fever prevailed in this locality as a distinct disease, but we now have a disease that suits well the description of typhoid fever given by Drs. Wood, Bartlett and other distinguished writers. Many of the symptoms of this fever are frequently presented in bilious remittent and pneumonia. One fact we have observed, is, that in localities where intermittent, remittent and pernicious fevers prevail *extensively*, we have less typhoid than in localities where there is but little miasmatic poison.

The disease we are about to describe does not appear to be contagious; even nurses do not seem to be in danger of contracting it. If this fact was fully established, by extended experience and observation, would it not aid materially in setting at rest the question of the non-identity of typhus and typhoid? That this disease is often confounded with typhoid pneumonia, and the typhoid grades of bilious fever, we have no doubt. We may have this adynamic or typhoid element in many diseases, but it would not be proper to pronounce all such diseases typhoid fever. We might enumerate many diseases that possess certain very prominent and important elements in *common*, while, at the same time, we have no difficulty in separating them into distinct individual species.

Dr. S. W. Thompson, in a very interesting communication, gives us the following description of a disease that prevailed last year in Clay County:

"Typhoid fever prevails far more extensively in this part of the State than it did a few years since. Indeed, this disease, so far as I can learn of surrounding physicians, who have been practising here for some years, was almost or entirely unknown until within a very brief period. Of late years, however, cases have occasionally shown themselves; but never so far, as I am aware, did it make its appearance in anything approaching to either an endemic or epidemic form until last summer and fall, when it, or a fever so closely allied thereto that the difference was to me inappreciable, prevailed so severely and so extensively in the town of Xenia as to deserve the term of endemic. It did not extend in this shape to the surrounding country, although many cases, perhaps a much larger number than in any one season before, occurred in my practice and that of other physicians.

"The prevalence of a fever of this kind in Xenia may perhaps be partly accounted for by the fact of a large amount of animal matter, 'the offal from a pork house,' being allowed to remain and decay there, so that the whole atmosphere seemed to be impregnated with the unwholesome and offensive effluvia arising therefrom.

"This view of the etiology of the fever is somewhat confirmed by the fact, that almost every case of sickness of any kind, even common remittent attacks, within the town, rapidly assumed an adynamic or typhoid grade or character.

"The situation of Xenia is on a high prairie, well supplied with good water, with no swamps, marshes or creek bottoms in its vicinity, and had hitherto enjoyed the character of being exceedingly healthy; I, therefore, can account for the visitation of a disease of this character upon no other ground whatever.

"The symptoms of these attacks were as follows: General malaise and loss of appetite, frequently accompanied by diarrhœa for several days before the patient complained of being unwell; dull headache in the extreme front or back part of the head; slight fever, with bad taste in the mouth, and excessive weakness and prostration came on, so that the patient was now compelled to take to his bed, reluctantly believing he was really ill.

"Upon being called at this time to see a person attacked in this manner, I usually found his condition to be as follows: Said he did not think he was much sick; had dull headache; bad taste in his mouth; felt very weak, and looked haggard; pulse about 100, small and weak, but thready; countenance looked dusky, surface rather hot; some diarrhœa, discharges yellowish green and very thin; tongue slightly coated with a whitish fur, edges red and fiery; (sometimes, however, this organ was very clean and red: such cases were invariably severe from the time the patient took to his bed until convalescence or death took place;) some pain in the back; urine deficient. Upon an examination of the abdomen, I found nothing unusual except in the right *Illiac fossa.* Upon pressure here, the patient would complain of slight pain, and upon putting the ear to this region, whilst pressure was firmly made, a gurgling sound could be distinctly heard. These symptoms continued with more or less intensity for a period of from four to eight days; about which time, the tongue would become dry and cracked, abdomen tympanitic and universally tender; stools frequent, usually retaining much the same character as at first; sometimes, however, they were very dark, and sometimes tinged throughout by blood, which was passed in great quantities in such cases; pulse small and thready, about 120; low, muttering delirium; *sordes* on the teeth; the patient continually desiring to get out of bed, in his efforts at which he would use all the violence of which he was capable. About the eighth day, *patechia*, and in some instances *vibices* made their appearance upon the abdomen and lower part of the chest and inside of the thighs; but the appearance of these was by no means constant, and in only a few instances could I discover sudamina. Profuse cold perspiration not unfrequently occurred at intervals throughout the attack, but I never noticed them to be indicative of a critical period in the disease. Retention of urine frequently occurred, and the latter stages, at which period dullness of hearing and a half comatose condition, with marked sub. *sultus tendinum* came on. *Decubitus* on the back. When a favorable change in the disease took place, the discharges from the bowels became less thin and frequent, fever less, pulse more slow and even, tongue began to clean off, and a warm but gentle perspiration was set up. The patient seemed to awake as from a troubled dream, often retaining no recollection of anything that had transpired since he was taken ill. The period at which this change took place was from the ninth to the twenty-first day."

It is vastly important, at an early period in this disease, to be able to distinguish it positively from a typhoidal state of the system; unless this can be done, we cannot arrive at a satisfactory diagnosis.

Every physician must desire to be able to distinguish this disease from all others, by evidence independent of lesions found after death. The foregoing are undoubtedly the characteristic symptoms of typhoid fever; and it seems where they are all present there is no possibility of mistaking the disease; but in the first few days after the access of the disease, many of the characteristic symptoms are not present, and a positive diagnosis cannot be made out.. The early symptoms, such as heat, thirst, loss of appetite, accelerated circulation, pain in the head and limbs, are common symptoms in many other febrile affections. Thus, the great and important diagnostic element in the treatment of this disease is doubtful, an element on a full knowledge of which alone depends the certainty of our therapeutical agents.

We have received a lengthy paper from our old friend, Dr. Benj. A. Allison, of Spencer, Indiana, and will present his description of typhoid fever in his own peculiar conversational style. He says in relation to this fever:

"The symptoms, as I have seen the disease, do not materially differ from those laid down by late standard authors; the peculiar eruptions, enlargement of the spleen, diarrhœas, bleeding at the nose, ringing in the ears, sudamina, and injected eyes, occurred in about half the cases I have treated. Frequency of the pulse disturbed and unrefreshing rest, headache or tightness across the forward, and tympanitic distention of the bowels, occurred oftener; but one of the most important considerations in this disease is to detect it in its early stage, so that the strength and vitality of the patient shall not be sacrificed in an attempt to speedily remove an affection that seems destined to have pretty much its own time and too often its own way. I have usually been tolerably successful in distinguishing this disease in its commencement, but, if I were called upon to fully explain the process by which I arrive at my conclusions, I might not in some instances be able to give more satisfaction than the *boys* do when they explain things by "*feeling it in their bones.*" The *absence* of symptoms, or rather of marked symptoms, is a leading trait of the first stage of this disease. And this is a point that should engage the physician's attention. I am tempted to try and commit to paper, as near as I can, the mode by which I would detect an attack of this fever in its first stage, and for this purpose I will summon to my aid the case of a worthy young man, whose untimely end was so regretted that all the circumstances of his sickness are fresh in my memory. The patient shall speak for himself. First visit.—"How long have you been sick, John?" "O, I can't say that I am sick; I have not been to say well for eight or ten days, but I've not been confined to my bed but little of the time." "Have you taken any medicine?" "A dose of pills three or four days ago." "Did they operate?" "Yes, very much, and my bowels have been a little loose ever since." "Any headache?" "No particular headache, but a feeling of lightness about the forehead." "Sleep sound and refreshing at night?" "Can't say that I do, I sleep pretty much all night but it don't appear to do me as much good as natural rest." "Any appetite?" "Don't know that I have—eat a little every day when they bring it to me, but could do just as well without it." "Bad taste in your mouth?" "Not very." "Let me see your tongue." Find it very slightly coated with a delicate white fur, with clean edges. "Any thirst?" "None of consequence." "Sweat any?" "Mother gave me some tea a day or two ago, after which I sweat some, but not any since, I believe." "Have any chills?" "No particular chills, but sometimes feel inclined to tuck the bed-clothes round my neck." "Any fever?" "Can't say that I have; feel little warm and restless at times." "Urine high colored?" "Not very." "Any difficulty in passing it?" "None." "Any pain or uneasiness in your bowels?" "I believe not." "Well, tell me now what pains or other symptoms you have that distress you most." "I have no particular pain or distress; I don't think much is the matter with me." Abdomen examined but no additional light elicited; his skin slightly sallow—a common symptom in nearly all the diseases of our locality; observed a little disposition to cough occasionally, and to take a longer breath than usual now and then.

Ascertained upon close inquiry that there were very obscure remission in the symptoms.

Now for the diagnosis. Here is a case without a single positive symytom. Is it slight functional derangement that afflicts him? Certainly not, for he has taken as much nourishment daily as some dyspeptics take who keep on foot, laboring and suffering ten times as much; and yet he is now confined to his bed, or, at any rate, cannot sit up without feeling more or less fatigued, and this debility is an important fact, for it cannot be reconciled with the rational and physical symptoms; neither have the discharges from the bowels been sufficient to produce it. So, here we will make our point.

Is it a case of marked intermittent fever? No reasons to believe it is, for there is too much uniformity in his feelings. Is it an obscure and mild attack of the remittent fever? It is still too uniform; besides it has not progressed fast enough; but yet the main point to be decided is whether it is of the latter variety of fever or a typhoid. The effects of medicines will be of considerable use to us here. We will give him pulv. Ip. comp. in 2 grain doses every two hours; enjoin him to remain quiet, with sufficient bed-clothes on him to make him feel comfortable; give him toast water as a drink, and between the second and third powder bathe his feet in hot water by placing the bathing vessel under the bed covers. If this course produces a gentle perspiration, with a quietness of the whole system, and a disposition to sleep, we will follow it with enough quinine to break up an ordinary intermittent. The course is carried out and we again visit him.

"How are you by this time, John?" "I don't think I'm quite as well as when you saw me yesterday morning; I was very quiet and moist when I commenced taking the second medicine you left, but after the second dose I became dry and little feverish, and havn't felt so well since."

Here is another point. Had it been a case of common remittent fever the quinine would have likely acted in conjunction with the first course, and would have prolonged and increased the perspiration to a copious sweat. But we will not follow the case through; sufficient to say that I pronounced it a case of typhoid fever on my second visit, and was more than suspicious of it at the start.

He died the twenty-ninth day after my first visit.

Dr. York, of Paris, Ill., does not think that typhoid fever has increased in this locality, but he occasionally meets with the disease. He firmly believes that the number of cases are multiplied by incorrect diagnosis, but does not point out the manner in which he distinguishes it from other analogous affections.

Dr. T. D. Washburn, of Lawrenceville, says that typhoid fever is on the increase in that county, at least in some localities, but in the town it has never gained a residence. The village is situated on the Embarrass river, with much low bottom land, a locality favorable for the prevalence of malarious diseases.

We will refer to some of the facts in relation to this disease, presented in the very able report of Dr. S. Thompson to the State Medical Society in 1856.

Dr. J. O. Harris, referring to Ottawa and vicinity, which it will be remembered is situated on the Illinois river, at the mouth of Fox river, necessarily a malarious district, says: "The cases called typhoid that I have seen have invariably lacked the characteristic features of that disease. Our fevers (as I view them), gradually assume a graver type from September to January, but always present unmistakable evidences of intermittent and remittent fever, and usually yield wholly or in part to the use of quinine or its equivalents. When treated according to the views above expressed they usually recover, but when treated as typhus or typhoid more than one-half die."

Dr. Vance who resides in Stark county, some distance from the main water courses of the county, says: "Typhoid fever has been a common disease among them for years." The doctor does not give the symptoms.

Dr. N. S. Davis, of Chicago, states that almost invariably typhoid fever steals upon its victim slowly. "He first feels dull and indisposed to active mental or physical exertion, his head feels dull, slightly aches or feels as if bound up tight, he has wandering pains in the back and limbs, occasionally liquid discharges from his bowels, his appetite is variable, and his sleep is often disturbed. These symptoms gradually increase for one or two weeks before the patient finally gives up and takes his bed, and when he does so his skin is dry but moderately hot, his pulse is 85 to 100 and less forcible than natural, all his secretions are diminished except from the mucus membrane of the illium, which generally gives rise to from one to six thin evacuations per day, his tongue is covered with a thick dirty white fur and red along the edges, his intellect is dull and disposed to wander when he is sleeping. The capillary circulation upon the surface is sluggish; in a word, all the organic actions are like the mind of the patient, dull and indifferent."

Dr. Rayburn, in his report to the American Medical Association, says: "Typhoid fever is on the increase in Missouri." The editor of the American Journal thinks the remark will hold good in almost every section of the United States. Dr. R. is of the opinion that in St. Louis this fever is superseding to some extent the epidemic billious fevers. This is also true in relation to the Wabash valley. We agree with Dr. R. that billious and typhoid fever are often so interfused as to render it almost impossible to determine which form of fever predominates. We have thought for several years that the two diseases oftentimes do exist in the system at the same time.

One fact we have noticed is, that in districts strongly impregnated with miasmatic poison, when we have typhoid symptoms, they are more *violent* at the commencement of the attack than in localities where there is comparative immunity from this poison, and the violent symptoms yield more readily than when they come on by a slow insidious process. When a patient gets sick by degrees, at the commencement but little evidence of disease, but a gradual getting worse, and in ten or fifteen days we have a delirium, subsultus, a dry cracked tongue, and other alarming symptoms, his chances of recovery are not as good as they would be were these symptoms present at the outset of the disease.

Your reporter has seen quite a number of cases within the last five or six years that are accurately described by Drs. Davis, Allison, and S. W. Thompson, while at the same time we have seen many cases of remitting fever in which a typhoidal element existed; but it is vastly important that we be able to distinguish this complication from the typhoid fever.

The diagnosis in enteric fever during the early stage of the disease is often attended with doubt and uncertainty. We may frequently avoid these errors by strictly observing the most characteristic symptoms of the disease at the beginning. Its mode of attack is generally slow and insidious with moderate diarrhœa, mental and muscular debility; soon afterwards we have a dull heavy expression of the countenance, face very red, bleeding at the nose, bronchial rale and cough, tongue white: in six or eight days we have a dry tongue, imperfect secretions, sudamina, rose-colored eruptions, tympanitus, deafness, stupor and delirium. In bilious fever, a disease with which it is most frequently confounded, we find yellowness of the skin, bilious vomiting, a distinct and regular remission of the fever, tongue brown, epigastric uneasiness, jaundiced urine; and if we have a typhoid element in the disease, to these symptoms are superadded dry and cracked tongue, subsultus tendinum, tympanitis, dullness of hearing, stupor and delirium.

From the symptoms above given it is very evident that in the beginning of the disease we should avoid a hasty decision, but at a more advanced period it seems that we have a group of characteristic symptoms that enable all observing physicians to make out a positive diagnosis.

TREATMENT.

Dr. S. W. Thompson remarks in his letter to your committee that his treatment is somewhat expectant. He says: "My ordinary treatment was to give, after the administration of some mild emetic, small doses of cal. ipecac and quinine every three or four hours, with cold sponging of the surface, and spirits of ether, nit. or spts. mindereri every two hours. In cases in which the nervous symptoms, manifested by subsultus, &c., &c., greatly predominated, or were very prominent, I gave the *mist. camphora.* in place of the above."

The *mist. camphora.* was, with me, a positive remedy during latter stages of the disease. The mercurial I continued no longer than gently to touch the gums, as evinced by a spongy condition of them, and an increased flow of saliva as shown by a moistened tongue, and subsequently reverted to its use as circumstances required. The ipecac I administered in as large doses as would be tolerated without producing emesis, and this, with the quinine, was persisted in nearly throughout the disease. Where great nervous irritability and restlessness existed I used the *pulv. dov.* with camphor as follows:

| | | | |
|---|---|---|---|
| Pulv. Dov., | - | gr. V. | every 2, 3 or 4 hours pro. re. rata. |
| Camphor, | - | gr. iss. ii. | |

In the early stages of the disease, to control the diarrhœa, I used some gentle astringent, as

| | | | |
|---|---|---|---|
| Plumb. Act. | - | gr. iii. | every 3 or 4 hours. |
| Tannin, | - - | gr. ii. | |
| Opium, | - - | gr. 1-4. | |

In the more advanced stages, however, I depended upon turpentine—gtts. x. or xx. every four hours—until its further use was contra-indicated by the occurrence of cystic irritation; when dangerous hemorrhage took place from the bowels I administered this remedy in half-drachm doses as often as circumstances seemed to require. In one case in which the patient discharged over a pint of blood every half hour for several hours, until she became almost bloodless, I gave it in ʒj doses every twenty minutes with entire success, and without any subsequent inconvenience. I must mention here, that apart from the use of turpentine in controlling the diarrhœa, in which I found it superior to all other remedies, I found it an invaluable remedy in overcoming the peritoneal tenderness and inflammation occurring in the latter stages of the disease now under consideration.

Allow me here to give you the treatment in full of one case, as, apart from its nature, there were peculiarities in the treatment instituted which may be of interest to others, as it was to me. The subject was a youth aged 16 years, previously in good health. The attack was at the commencement such as I have before described, and in spite of treatment progressed from bad to worse till the fourteenth day, at which time his condition was as follows:

*Case.*—Constant fever, pulse 120 small and thready but rather hard, frequent discharges from the bowels, abdomen excessively tympanitic, considerable peritoneal tenderness and gurgling upon pressure in right *Illiac fossa;* he is drowsy and lies with his eyes only half closed, is with difficulty aroused, low muttering delirium with hurried respiration, when aroused constantly desired to get out of bed, subsultus tendinum with tongue red at edges but the center and back part dry, deeply fissured and covered with a brown coat, sordes on teeth. Profuse perspiration would occasionally take place. As the disease had resisted all ordinary remedies and death seemed inevitable, I determined, without any sanguine hopes of the result, to try the sulph. quinine in full doses. (At this time I had not seen anything recommending such a course in the advanced stages of typhoid fever, but I shortly afterward found that I had overlooked a communication on this very subject, published in Braithwait's Retrospect, No. 28, page 22).

I accordingly gave as follows:

Quinine, ℈jii.

Potassa Nit., ℈ji. } Cht. No. viii.; 3 hours apart.

Ipecac, gr. x.

With turpentine, gtts. xx., between each powder. The first dose or two produced some nausea, but not to the extent of emesis. In twenty-four hours his condition was much improved, as follows: Tongue moist and not so fiery at edges, less drowsy but sleeps quietly, pulse 100 soft and full, fever much less. As symptoms of cinchonism now appeared, I desisted from the use of quinine, continuing the other remedies with mist. camph. At the expiration of six hours, however, the unfavorable symptoms again made their appearance, and I recommenced the use of the quinine, and this time was enabled to desist from it much sooner. I was, at the end of twelve hours further, induced to use it again, but this time three doses as above sufficed, and convalescence immediately set up, which, with the assistance of wine, &c., was far more rapid than could have been anticipated. Since then I have instituted the above treatment in several bad cases of typhoid fever, and the result has been encouraging.

In this description of the fever now under consideration I have spoken of and described the most aggravated cases; and in speaking of the treatment I have given but the leading features, thinking not to weary you still further by uninteresting details. Some few cases seemed to be stopped in their outset by the abortive treatment with large doses of quinine; but the premonitory and even commencing symptoms of typhoid fever are not sufficiently well understood, in my opinion, to make a diagnosis absolutely certain and positive at this early stage.

Dr. B. A. Allison says: "After I have made up my mind that it is a case of typhoid fever I have to deal with, I pursue a cautious unoffending course. But as I have no specific, nor any favorite course of treatment to which I attach particular importance I will merely make mention of some remedies that I have at least had no cause to complain of:

"An occasional mild laxative, such as rhubarb, senna, magnesia, oil with a little turpentine, or small portions of calomel rubbed up with gum arabic, warm bath (always administered in the horizontal position), particularly in cases where there is no latent affection of the lungs or bronchia, frequently sponging the surface with tepid or cool water, as is most agreeable, and when there is considerable restlessness, with wandering pains or uneasiness in the limbs, I use alcoholic spirits instead of water; would be sparing of spirits, however, if there was considerable cerebral disturbance; an occasional course of pulv. ip. comp. in small portions, light poultices to the abdomen, camphor water, Hoffman's anodyne, turpentine emulsion, &c., &c. In the latter stages, where the patients have been low, I have used externally all over the surface an infusion of cantharides in spirits of turpentine. In cases where the bowels appear to be the focus of morbid action, I use anodyne and mucilage injections; also use in certain cases nitrate silver, sulph. zinc or sulph. copper by injection. I lay as much stress on the nursing as anything else; and for the rules to govern the physician in this department, I would advise a perusal of Armstrong's practice, an old work, out of date, but yet containing some of the best general directions for managing or nursing low forms of fever that I have met with."

Our own experience does not favor to any considerable extent the use of quinine in this disease. If it is complicated with bilious remittent, quinine may be given with advantage. We usually begin the treatment with a moderate mercurial purge; blue mass is the best. After it operates, if there is remission in the fever, we give a few doses of quinine, opium and ipecac. We use pretty freely senega tea, and sponge the body frequently with saleratus water. In two or four days, if the tongue is dry, we give minute portions of calomel, ipecac and opium every two hours for twelve or twenty-four hours; after which we use oil and turpentine to move the bowels. When the pulse is frequent we combine with the powder, cimicifuga racemose with the most happy effect. After repeating the calomel powders once or twice, if the tongue continues dry, we use the turpentine in a solution of gum arabic, x. or xii. drops every three or four hours. For subsultus we prefer ammoniated tinct. of valerian. We use turpentine externally and hop fomentations. For diarrhœa we find opium and acetate lead the best remedies. In the latter stage we use wine freely, and frequently some tonic infusion, such as camomile or cinchona. We keep up the temperature of the surface with hot bricks and mustard plasters. The disease cannot be aborted, and it is desirable for the physician to husband the strength of his patient. We have never seen a case where we thought bleeding would be admissable. Blisters to the abdomen are not beneficial; but frequently, when the delirium is great, they exert a good effect when applied to the nape of the neck. Our treatment is designed to meet the symptoms with the best known remedies, without even a hope or expectation of aborting the disease. A remedy that will arrest it may exist in nature, but it is left for future observation and experience to point it out. We have tried the veratrum viride in a few cases where the pulse was 130 to 150. The remedy diminished the frequency of the pulse, but did not arrest the progress of the disease; but still we have reason, from our little experience in its virtue, to hope that it will prove a valuable agent in the grave form of this affection, and all other diseases where it is desirable to control the action of the heart.

The important indications in the treatment of this disease are to have free ventilation of the room, keep the patient clean, nurse well, allay excitement, prevent the development of local inflammation, and at the same time support the patient's strength.

### DYSENTERY.

For the last two years your reporter has not seen many cases of this disease. In the autumn of 1851 it prevailed in an epidemic form in the vicinity of Prairieton, about twelve miles from this place. About one-half of those attacked with the disease died. The fatality was greater among infants than adults. The symptoms were pain in the bowels, frequent bloody mucous discharges, amounting sometimes to ten or twelve per hour, tenesmus, fever, frequently bilious coat on the tongue; in the advanced stage the stools had a peculiar odor; tympanitis and great pain on pressure over the track of the colon; the stools in the last stages resembled the washings of flesh. The treatment adopted by the physicians in that locality was principally mercury and quinine. They considered it connected with the ordinary bilious fever, and produced by the same cause. In the fall of 1852 the same disease visited this side of the river, about ten miles south of Marshall, in a malarious district, and in the district where it prevailed in 1851 they had no cases. Your reporter visited many of these cases, and found by experience that the opiate treatment is absolutely necessary for the successful management of this disease. Under no circumstances, after the first two or three days from the access of this disease, could we be induced to administer cathartic doses of mercury, and we have doubts in relation to the propriety of giving it in minute doses. Our chief reliance was in opium and saline cathartics, with injections of acetas plumbi and morphine, to relieve the distressing tenesmus.

In 1853 the epidemic visited Marshall, and we found no cases in the localities where it had prevailed the two preceding years. Many children died with the disease, but there were but few deaths in persons over the age of six years. The cause of this we attributed to the fact that in children we could not administer the opium in sufficiently large doses to control the inflammation.

In 1854 the disease prevailed north and west of this place, and presented very much the same character that it did here and south of us, and we adopted the same course of treatment.

In the fall of 1855 the same disease prevailed at Paris, Ill., sixteen miles north of Marshall, and we have a very interesting account of it published in the "*North Western Medical Journal*," of July, 1856, by Henry W. Davis, M. D.

The doctor attributes the cause of the disease to malaria. He says: "It not only appears to be completely under the sway of miasmatic poison, but it may be said to be the direct effect of it." Our experience is not very favorable to the doctor's theory. We are not prepared to say that malaria has any particular agency in the production of this disease. It may exist in connection with intermittents or remittents, but if the disease is produced by miasma, how will we account for its migratory character? Since 1853 we have hardly had a case in this locality, while at the same time we have had, at least in one season, a superabundance of malaria. If the disease is produced by this cause, why is it not a common disease in the Wabash valley?

It may be proper to state that in some cases of this affection, where there was an intermission in the fever and other symptoms indicating its connection with our ordinary bilious fever, we found quinine beneficial. Your reporter had a severe attack of the disease, and had a good opportunity of testing the efficacy of opium as a remedy, and he feels confident that without it he would not have survived. We look upon it as a *sine qua non*, in the treatment of this distressing and grave affection.

